# Relationship between Brain Natriuretic Peptide and Recurrence of Atrial Fibrillation after Successful Electrical Cardioversion: an Updated Meta-Analysis

**DOI:** 10.21470/1678-9741-2017-0008

**Published:** 2017

**Authors:** Xiangdong Xu, Yongqing Tang

**Affiliations:** 1 Central Hospital, Jiading District, Shangai, China.

**Keywords:** Atrial Fibrillation, Atrial Flutter, Electric Countershock, Natriuretic Peptide, Brain

## Abstract

**Objective:**

To investigate the relationship between brain natriuretic peptide and recurrence of atrial fibrillation after successful electrical cardioversion.

**Methods:**

Medline and Embase databases were used to identify publications evaluating BNP/N-Terminal (NT)-proBNP levels in association with atrial fibrillation recurrence after successful electrical cardioversion. Nineteen studies that fulfilled the specified criteria of our analysis were found.

**Results:**

Baseline BNP/NT-proBNP levels of the atrial fibrillation recurrence group were significantly higher than those of the sinus rhythm maintaining group (SMD -0.70, CI [-0.82, -0.58]).

**Conclusion:**

Our analysis suggests that low BNP/NT-proBNP levels are associated with sinus rhythm maintenance, and baseline BNP/NT-proBNP concentrations may be a predictor of atrial fibrillation recurrence after successful electrical cardioversion.

**Table:** 

Abbreviations, acronyms & symbols	
AF	= Atrial fibrillation
BNP	= Brain natriuretic peptide
LVEF	= Left ventricular ejection fraction
SD	= Standard deviation
SR	= Sinus rhythm

## INTRODUCTION

Atrial fibrillation (AF), the most prevalent cardiac arrhythmia, is a costly disease associated with significant morbidity and mortality. There are two modalities of treatment for AF: rate control and rhythm control. Rhythm control is preferred in cases in which sinus rhythm (SR) is expected to be maintained after cardioversion. Direct-current cardioversion is frequently used to restore SR in patients with AF, but the recurrence rate of AF after direct-current cardioversion is 50% after six months^[1]^. There are many predictors of SR after cardioversion of AF, of which BNP/NT-proBNP is a commonly described one.

Evidence from several studies supports the relationship between BNP/NT-proBNP and AF recurrence before electrical cardioversion, but other studies have failed to find an association. In 2011, a meta-analysis by Tang et al.^[2]^, which included 10 trials and 618 patients, concluded that higher plasma BNP levels are associated with a greater risk of AF recurrence. Since 2011, nine additional randomized controlled trials were published with double the sample size and that contained information on the association between BNP/NT-proBNP and AF recurrence.

Under this circumstance, this updated meta-analysis was conducted to reveal a wealth of evidence and provide more valid information for clinical practice.

## METHODS

### Search Strategy and Selection Criteria

This analysis was performed according to the guideline of the Meta-analysis of Observational Studies in Epidemiology Group^[3]^. Medline and Embase databases were used to identify relevant publications indexed between January 1980 and August 2016, using the following key words ["natriuretic peptide" and "atrial fibrillation"] or ["atrial fibrillation" and "cardioversion"] or ["atrial fibrillation" and "electrical cardioversion"]. We reviewed abstracts except for articles that focused on natriuretic peptide in AF cardioversion, for which we obtained full text. A review for the references of identified studies was conducted and limited the language to English. The corresponding authors were contacted if further unpublished data were needed. And a manual search was also conducted for relevant review articles and for abstracts of the scientific sessions of the American College of Cardiology, the American Heart Association and the European Society of Cardiology for the past five years.

Studies were considered eligible for inclusion in our meta-analysis if they meet the following criteria: (i) they were observational studies; (ii) they evaluated the potential relationship between plasma BNP/NT-proBNP levels before cardioversion and AF recurrence after successful electrical cardioversion; (iii) they used AF recurrence rates as an index of the outcome; and (iv) they used a follow-up period of ≥ 5 days. The 5-day follow-up was decided on the basis of the 2010 European Society of Cardiology guidelines, which indicate a late recurrence of AF that occurs more than five days after successful electrical cardioversion^[4]^.

### Assessment of Study Quality

To examine biases in the studies and their effects, quality assessments according to the following criteria were conducted: (i) whether the inclusion criteria and the end of the event were clearly defined; (ii) whether the follow-up period was adequate; (iii) whether all cases were followed up (*i.e.*, withdrawal rate was <20%); (iv) whether confounding and predicting factors were distinguished; and (v) whether there was a clear description of the detection system used to identify the incident endpoint.

### Data Extraction

Two reviewers independently extracted data from each study. Results were compared, and any disagreements were resolved by consensus. The extracted data included first author, patient characteristics (*e.g.*, left ventricular ejection fraction (LVEF), AF duration, follow-up period), AF detection methods, number of AF recurrence and SR-maintaining patients, and mean and standard deviation (SD) of BNP/NT-proBNP concentrations in the AF recurrence and SR-maintaining groups. If the documents provided medians and range, the method of Hozo et al.^[5]^ was used to calculate the mean and SD.

### Statistical Analysis

Data were collected and analyzed using STATA software (version 12.0 STATA Corp., College Station, TX, USA). Cochrane's Q test is the most commonly used method to evaluate heterogeneity. In our article, the result of this test is shown by I^2^, because the I^2^ statistic describes the percentage of variation across studies that is due to heterogeneity rather than due to chance. I^2^ = 100% x (Q-df )/Q. I^2^ is an intuitive and simple expression of the inconsistency in studies' results. We considered an I^2^ statistic of 50% or more as indicative of a considerable level of heterogeneity; if I^2^ >50%, the random effects model was used^[6]^. Otherwise, the fixed effect model was used. We planned to perform sensitivity analysis and subgroup analysis to explore the potential influence of heterogeneity.

## RESULTS

### Literature Search

**Fig. 1:**
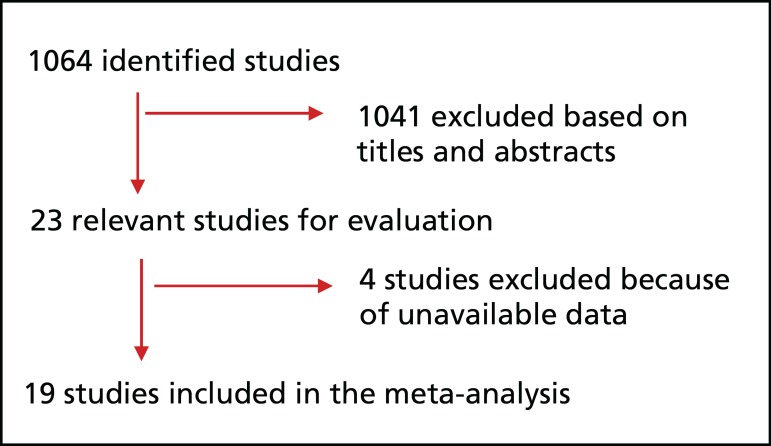
Meta-analysis flowchart.

Our initial literature search identified 1,064 studies (Figure 1), of which 19 were selected in our analysis^[7-25]^. A total of 1,373 patients were enrolled in these studies: 657 cases were classified in the AF recurrence group and 716 in the SR-maintaining group. Basic characteristics of the 19 studies are shown in Table 1.

**Table 1:** Basic characteristics of the included studies.

Study	Study characteristics	AF duration	N	AF recurrence rate	Follow-up period	AF detection	Detection index
Lewicka et al.^[7]^	preserved LVEF 55±5%	median 51 days	52	92%	12 months	pacemaker logs	BNP
Andersson et al.^[8]^	10% patients with heart failure	>7 days	199	54%	1 month	ECG	NT-proBNP
Mukherjee et al.^[9]^	preserved LVEF 56.78±140.6%	<1 year	82	44%	12±2 weeks	Physical, ECG	NT-proBNP
Govindan et al.^[10]^	preserved LVEF>50%	<18 months	54	61%	12 months	Holter, ECG, physical	NT-proBNP
Kawamura et al.^[11]^	preserved LVEF 61.1±9.6%	37±26 days	142	38%	24 months	ECG	BNP
Barassi et al.^[12]^	preserved LVEF 58.7±6%	3 months	57	33%	3 weeks	ECG	BNP
Falcone et al.^[13]^	NYHA I or II	93±65 days	93	28%	6 months	Holter, ECG	BNP
Wozakowska-Kaplon et al.^[14]^	NYHA I, LVEF 58.1±6.4 %	>48 hours, <12 months	77	39%	6 months	ECG, Holter	BNP
Kallergis et al.^[15]^	LVEF 57.1± 8.5%	>3 months	40	23%	1 month	ECG	NT-proBNP
Ari et al.^[16]^	preserved LVEF 55.5±2.98%	23.2±6.5 months	58	34.5%	6 months	ECG	BNP
Mollmann et al.^[17]^	LVEF >45%	181.5±327days	49	36%	4 weeks	Holter, ECG	NT-proBNP
Lombardi et al.^[18]^	LVEF >45%	1 month-1 year	53	34%	3 weeks	Holter, ECG	NT-proBNP
Tveit et al.^[19]^	NHYA I	median 10.5 weeks	129	69%	6 months	ECG	NT-proBNP
Buob et al.^[20]^	LVEF 57±11%	90±75 days	25	44%	4 weeks	Physical, ECG	NT-proBNP
Shin et al.^[21]^	normal LVEF	10.9±8.3 weeks	34	29.4%	11 days	ECG	NT-proBNP
Lellouche et al.^[22]^	NYHA I or II	3.7 months	66	45%	1 year	ECG	BNP
Mabuchi et al.^[23]^	NYHA II or III	mean 5.8 months	65	45%	553 days	Physical, ECG	BNP
Beck-da-Silva et al.^[24]^	NYHA I or II, preserved LVEF	6 months	14	36%	2 weeks	ECG	BNP
Watanabe et al.^[25]^	NYHA I -III, LVEF 59±10%	(1-350) days	84	76%	12 months	Holter, ECG	BNP

AF=atrial fibrillation; BNP=brain natriuretic peptide; ECG=electrocardiogram; LVEF=left ventricular ejection fraction; NYHA=New York Heart Association

**Fig 2:**
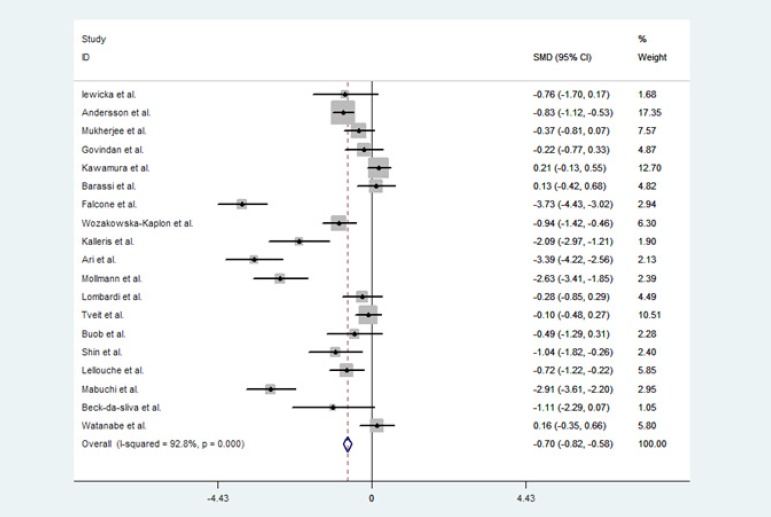
BNP/NT-proBNP levels and AF recurrence: an updated meta-analysis.

Findings from the meta-analysis related to the relationship between plasma BNP/NT-proBNP level and AF recurrence after successful electrical cardioversion in patients with AF are shown in Figure 2. When data were pooled across studies, the baseline BNP/NT-proBNP levels in the AF recurrence group were significantly higher than those in the SR-maintaining group (SMD -0.70, CI [-0.82, -0.58]).

**Fig. 3:**
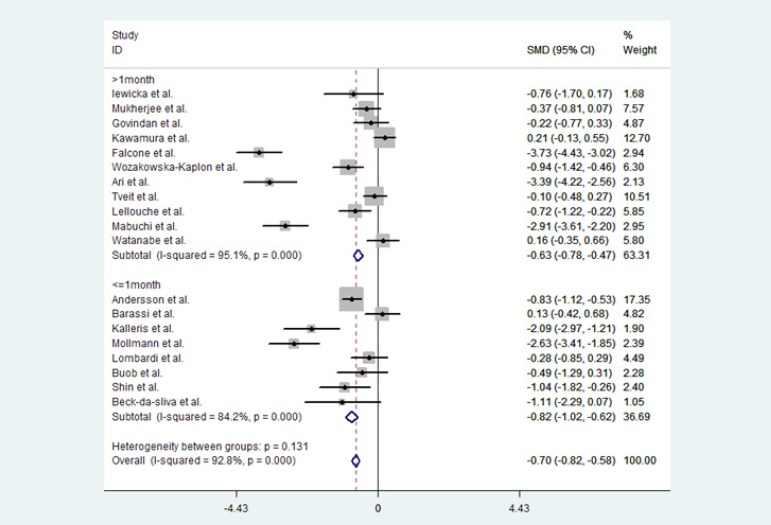
BNP/NT-proBNP levels and AF recurrence: subgroup analysis.

Evidence of significant heterogeneity was observed (*P*<0.0001; I^2^=92.8%). We analyzed the heterogeneity using the random effects model. To explore potential reasons for heterogeneity, we conducted a subgroup analysis to investigate the effect of follow-up duration on BNP/NT-proBNP levels between the SR and AF groups, because the duration of the follow-up period was a major difference in the included studies. This subgroup analysis showed in the long-term follow-up subgroup (defined as followup >1 month) (SMD -0.63, CI [-0.78, -0.47]) and the short-term follow-up subgroup (defined as follow-up of ≤ 1 month (SMD -0.82, CI [-1.02, -0.62]), baseline BNP/NT-proBNP levels in the AF recurrence group were significantly higher than BNP/NT-proBNP levels in the SR-maintaining group (Figure 3). This indicated than BNP/NT-proBNP concentrations in the AF recurrence group were significantly higher than those in the SR-maintaining group.

**Fig. 4:**
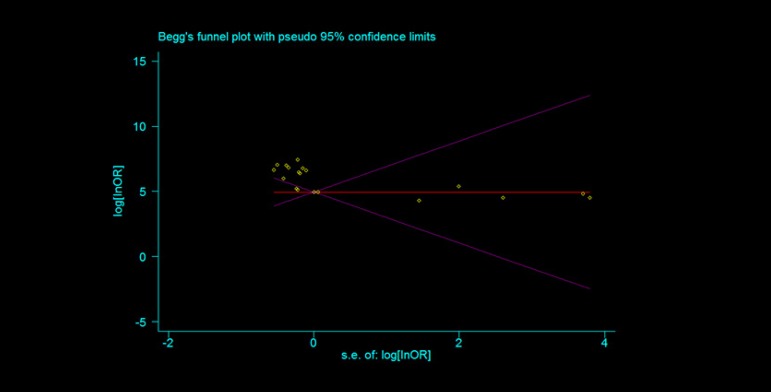
Begg's funnel plot.

When the study with the shortest follow-up^[21]^ was discarded, the sensitivity analysis produced results similar to those obtained when this study was included (SMD -0.69, CI [-0.81, -0.57]). The heterogeneity was observed in our analysis, which may be attributed to differences in patient characteristics, such as etiology, AF duration, age, and heart function. Possible language and publication biases may also be considered. Distribution of data points on a funnel plot (Begg test) indicated that bias were possibly related to language and publication bias (Figure 4).

## DISCUSSION

Our meta-analysis aimed to further explore the relationship between BNP/NT-proBNP levels before successful electrical cardioversion in patients who maintained SR and who had AF recurrence during the follow-up period. The result of our analysis showed that patients with lower BNP/NT-proBNP concentrations were more likely to remain in SR after successful electrical cardioversion. This analysis was conducted in 2011, when 10 studies were included in our analysis. Our present analysis contains nine additional studies, including studies with negative results and twice the number of patients. A previous meta-analysis by Zografos et al.^[26]^ was conducted. In contrast to their meta-analysis, we pooled the BNP/NT-proBNP results. Moreover, the studies involving both electrical cardioversion and pharmacologic cardioversion were excluded.

B-type natriuretic peptide, a neurohormone, is synthesized as a prohormone in the heart in response to increased myocardial wall stress from volume or pressure loading and other factors. The peptide proBNP is cleaved into BNP and the biologically inactive fragment NT-proBNP. BNP is secreted primarily by ventricular myocytes. In the patients in our analysis, which mainly have preserved ejection fraction, high BNP/NT-proBNP level may have occurred for a different reason. In patients with AF, there is evidence that BNP is secreted by the atrium. Tuinenburg et al.^[27]^ studied atrial BNP gene expression in patients with paroxysmal and persistent AF and found that the atrium was the major site of BNP gene expression. Silvet et al.^[28]^ found that the increase in BNP levels after atrial stretching and volume overload was closely related to chronic AF. In addition, pathologic changes such as the enlargement and fibrosis of the atrium may also cause increased BNP^[29]^. BNP levels may also reflect a higher degree of systematic inflammation, which is consistently associated with AF^[30]^. The question of whether BNP/NT-proBNP can be used as an effective indicator in the proactive prevention of AF or its recurrence or in the likelihood of success of cardioversion treatment has, therefore, attracted wide attention.

The present updated meta-analysis of 19 AF-related publications^[7-25]^ showed that after successful electrical cardioversion, AF was more likely to recur in patients with high, rather than low, plasma BNP/NT-proBNP concentrations. This indicates that plasma BNP/NT-proBNP level is a predictor of AF recurrence. Our subgroup analysis achieved the same result, regardless of the long- and short-term follow-up period, which indicated that the baseline BNP/NT-proBNP concentrations may be a predictor of AF recurrence after successful electrical cardioversion. Distribution of data points on the funnel plot graph after application of the random effects model indicated possible language and publication biases.

As a biomarker indicator, plasma BNP/NT-proBNP level has the advantages of cost- effectiveness, convenience, and a high degree of reliability^[31]^. The measurement of plasma BNP/NT-proBNP levels may help predict the risk of AF recurrence, thus helping the initial selection of suitable patients for AF treatment.

Our analysis suggests that low BNP/NT-proBNP levels are associated with SR maintenance, which is consistent with our previous finding even after including nine additional studies and doubling the number of patients. Even though using BNP/NT-proBNP levels could not guide electrical cardioversion by current evidence, our work's purpose is to suggest that BNP/NT-proBNP levels may help predict the risk of AF recurrence, thus helping the initial selection of suitable patients for electrical cardioversion; therefore, further research is needed to explore such a cutoff of BNP/NT-proBNP value to select the right patients for electrical cardioversion.

### Study Limitations

A limitation of this study is the significant heterogeneity, which probably reflects several differences between the included studies, such as patient characteristics. Nevertheless, we failed to observe a significant difference between studies with short- long-term follow-up. Another potential factor contributing to the observed heterogeneity is the difference in patient characteristics, such as etiology and ejection fraction. We included patients with AF with varying etiology; details of the etiology were not always available, even though the majority of studies included patients with preserved LV systolic function, and details on diastolic or left atrial function were not always available. Differences in these variables may account for the observed between-study differences as well as the significant in-study variation, which is evident in the wide SD of BNP or NT-proBNP levels observed in many studies.

**Table:** 

Authors' roles & responsibilities	
XX	Conception, acquisition, analysis, interpretation of data, work review; final approval of the version to be published
YT	Conception, acquisition, analysis, interpretation of data, work review; final approval of the version to be published
